# Letter IV

**Published:** 1804-11-01

**Authors:** 


### LETTER IV.

### OF QUACKS AND EMPIRICISM.

IT may appear a matter of surprize to some of your readers, that Germany should furnish so large a contingency of doctors, to improve the constitutions of the English; lish; whilst it may be admitted, that the continent does not afford more prolific sources of impudence than our home manufactories; but the phenomenon will not appear wonderful when it is considered, that no empiric is allowed to exhibit a single nostrum in Germany, which, of course, encourages a greater importation into this country, in which luxury and money are abundant, and consequently enervated constitutions, with an increased avidity to procure their restoration, become prominent features of a luxurious nation.

The state physicians in Vienna act with a dignity and decision unknown here; and where the sovereign head of the empire co-operates with them, in preventing the mischiefs resulting from empiricism, whilst this country opens a ready market for medical fraud, which at the same time is sanctioned by persons in power, for the sake of the revenue arising from patents, an object of higher estimation than the health of the community. In the distinguished period of the late Baron Van Swieten, empiricism never reared its fascinating and destroying head; and my honoured friend, Baron Quarin, told me, that it never would be tolerated; even Mesmer, who made some slight attempts to practise his deceptions in Vienna, escaped by an hasty flight the restraint of a prison; he found, however, an asylum among the cognoscenti and devotees in Paris, where he made more dupes as well as more money, than was effected by his pupil Demanaduke, by Perkins the American, Benamor the Algerine in London, and others who have varied the mode of magnetism; but whose metallic tractors and operations uniformly possess a polarity of attraction to the metallic substances in English pockets. I shall, however, at present dismiss this immense phalanx of Adventurers, who indeed possess a species of character too interesting to be allowed to sink into oblivion, and are truly worthy of being recorded in the annals of empiricism. At the same time I confess, it is not decorous to mention these in the page that contains the names of the illustrious Van Swieten, Quarin, and Ingenhoutz. The amiable and philosophic Ingenhoutz, now I believe deceased, told me, what no other physician ever could say, that he had under his medical care at one time *thirteen* imperial patients; and Quarin, who directs the first hospital in the world, with a spirit becoming his high character, as the physician and friend of the late Emperor Joseph, said to him in his last illness,

"Sire,

"Sire, in twenty-four hours your subjects will lose the best of Princes." Let it be recorded to the glory of Joseph, that he immediately gave Quarin a title of nobility and a pension of about £. 2000 a year, as a reward of his integrity and merit.

These are anecdotes, which I introduce as likely to entertain some readers, as well as to prove, that whilst I exhibit so many German impostors, I would devote with infinitely more pleasure the tribute of applause to every illustrious character on the Continent; and however great the chasm, I must now sink into a detail of others, whose consequence results from a source as distant from the other's as the poles.

### Character IV. Dr. Lamert.

A remarkable concatenation of circumstances has distinguished the origin and progress of twin characters in ancient, as well as in modern times; the latter of which, alone become objects of our present contemplation. From the school of Edinburgh, raised by the first Monro, as from the Trojan Horse, issued an host of heroes; at the same period, a Hunter and a Fothergill, in London; a Cleghorn in Dublin, and a Russell in Aleppo, did honour to their illustrious master. About the same eventful time, a Johnson, the first moral writer, and a Garrick, the first actor, centred, almost as exiles, in the metropolis. So the celebrated Lackington the bookseller, and Lamert the empiric, were twin adventurers; the first, from being an infidel, centred into a mystic devotionalist; and the latter has exhibited no less eccentricity. By birth a German, he found an asylum, as a servant, in the house of Mr. Goldsmith, of Thavies Inn; but, to mend his fortune, when Lackington rented a little shop in Chiswell Street, Lamert rented the next door, where he sold chalk balls to whiten buttons and buckles of a silvery hue. He arrived at such perfection in this art, as for a few pence he would silver over the whole paraphernalia of a suit of cloaths, by which he raised as much money as enabled him to commence a professional doctor. His first outset was a composition of spirit of wine, sweetened and coloured with treacle, which he entitled Swietzer's balsam, from the name of Switzer, in Germany, the place, or the vicinity of the place, from whence this illustrious character originated; and to give efficacy to his medicine, he assumed the title of Dr. Lamert.

This

This self-created Doctor, although he pretended to cure, infallibly, almost every disease incident to the human body, would have lived and died unnoticed, were it not from the reflected lustre of having been the patron of the celebrated Brodum; so, Bucephalus would never have been recorded in history, had he not possessed the honor of carrying the conqueror of the world, Alexander the Great. If the Macedonian hero claim immortality from his wonderful prowess by *land;* Lamert, with equal assurance, lays claim to prowess by *water,* for he delicately insinuates in his bills, "that in any disorder incident to the human body, and the afflicted know not the real disorder, or what it proceeds from, by bringing or sending their morning urine, may depend on having their disorder really informed them, *as the Doctor's admirable knowledge from the urine has established his fame.*"

With the gallantry of his respectable counter-part, Dr. Day, already noticed, he has introduced a postscript, to inform "any lady or gentleman, that he accommodates them with genteel lodgings at his house."

It must, however, be confessed, that a vein of piety is assumed in many of his advertisements, and the sacred name is introduced to sanction deceit, and cover ignorance, whilst he impudently claims "the blessing of God" on the cure of a venereal nose; and by the "intervention of providence," in curing an ulcer in the thigh, intimates that he is "the agent of the Almighty."

It must be painful to every mind of religious sentiment, to observe the levity, if not impiety, of introducing the sacred name to sanction imposition, a conduct indeed of frequent notoriety. To render medicines more successful, or at least so to impress it on the patient, already weakened by disease, and credulous by hope, the Deity is invoked by "*praying doctors,* to bless the means;" and thus, by a miracle, to suspend the operations of nature. Men assuming so high a function, acquire a consequence that merits a distinguished place in your valuable repository, where the names next to be recorded, are Dr. Gardner, and Dr. Benamor. But as Dr. Lamert has occupied so large a portion of your valuable miscellany, I am induced to postpone the history of the illustrious Brodum, as highly worthy of singly occupying a niche, and as exhibiting one of the most brilliant constellations in the galaxy of empirical impostors, commemorated by

IATROS.

